# Transition to a mesenchymal state in neuroblastoma may be characterized by a high expression of GD2 and by the acquisition of immune escape from NK cells

**DOI:** 10.3389/fimmu.2024.1382931

**Published:** 2024-04-26

**Authors:** Sabina Di Matteo, Maria Teresa Bilotta, Andrea Pelosi, Dorothee Haas, Tobias Theinert, Gerrit Weber, Paul-Gerhardt Schlegel, Matthias Berg, Lorenzo Moretta, Enrico Maggi, Bruno Azzarone, Paola Vacca, Nicola Tumino, Ignazio Caruana

**Affiliations:** ^1^ Tumour Immunology Unit, Bambino Gesù Children’s Hospital Istituto di Ricerca e Cura a Carattere Scientifico (IRCCS), Rome, Italy; ^2^ Innate Lymphoid Cells Unit, Immunology Research Area, Bambino Gesù Children’s Hospital Istituto di Ricerca e Cura a Carattere Scientifico (IRCCS), Rome, Italy; ^3^ Department of Paediatric Haematology, Oncology and Stem Cell Transplantation, University Hospital of Würzburg, Würzburg, Germany

**Keywords:** neuroblastoma, ganglioside sialic acid-containing glycosphingolipid-2, NK cells, immunotherapy, primary tumor cells

## Abstract

**Background:**

Neuroblastoma (NB) is characterized by both adrenergic (ADRN) and undifferentiated mesenchymal (MES) subsets. The ganglioside sialic acid-containing glycosphingolipid (GD2) is widely overexpressed on tumors of neuroectodermal origin promoting malignant phenotypes. MES cells are greatly enriched in post-therapy and relapsing tumors and are characterized by decreased expression of GD2. This event may cause failure of GD2-based immunotherapy. NK cells represent a key innate cell subset able to efficiently kill tumors. However, the tumor microenvironment (TME) that includes tumor cells and tumor-associated (TA) cells could inhibit their effector function.

**Methods:**

We studied eight NB primary cultures that, in comparison with commercial cell lines, more faithfully reflect the tumor cell characteristics. We studied four primary NB-MES cell cultures and two pairs of MES/ADRN (691 and 717) primary cultures, derived from the same patient. In particular, in the six human NB primary cultures, we assessed their phenotype, the expression of GD2, and the enzymes that control its expression, as well as their interactions with NK cells, using flow cytometry, RT-qPCR, and cytotoxicity assays.

**Results:**

We identified mature (CD105^+^/CD133^−^) and undifferentiated (CD133^+^/CD105^−^) NB subsets that express high levels of the MES transcripts WWTR1 and SIX4. In addition, undifferentiated MES cells display a strong resistance to NK-mediated killing. On the contrary, mature NB-MES cells display an intermediate resistance to NK-mediated killing and exhibit some immunomodulatory capacities on NK cells but do not inhibit their cytolytic activity. Notably, independent from their undifferentiated or mature phenotype, NB-MES cells express GD2 that can be further upregulated in undifferentiated NB-MES cells upon co-culture with NK cells, leading to the generation of mature mesenchymal GD2^bright^ neuroblasts. Concerning 691 and 717, they show high levels of GD2 and resistance to NK cell-mediated killing that can be overcome by the administration of dinutuximab beta, the anti-GD2 monoclonal antibody applied in the clinic.

**Conclusions:**

NB is a heterogeneous tumor representing a further hurdle in NB immunotherapy. However, different from what was reported with NB commercial cells and independent of their MES/ADRN phenotype, the expression of GD2 and its displayed sensitivity to anti-GD2 mAb ADCC indicated the possible effectiveness of anti-GD2 immunotherapy.

## Introduction

The ganglioside sialic acid-containing glycosphingolipid 2 (GD2) displays mild restricted expression in the human normal cerebellum, neurons, skin melanocytes, and peripheral pain fibers. However, it is widely overexpressed on several tumors of neuroectodermal origin, including human neuroblastoma (NB) and melanoma (1). Although GD2 appears to participate in cell signaling, its function in normal cell physiology has not been fully elucidated (2). In cancer, GD2 promotes malignant phenotypes such as increased cell proliferation, growth, migration, and invasion activity (2–6). Due to these properties and its overexpression, it proved to be an excellent candidate for the development of immunotherapeutic approaches, particularly in the NB setting. The development and clinical translation of a monoclonal antibody directed against GD2, dinutuximab beta, has revolutionized the clinical outcomes of NB patients, now being a conventional treatment for these patients (1–3). However, even though this treatment marked a significant improvement in event-free survival and overall survival (OS), 40% of the patients still relapsed (3). Therefore, new and more innovative strategies are in the development stage. The low or even absent expression of major histocompatibility complex I (MHC-I) favored the development of MHC-I unrestricted tumor cell strategies, mainly exploiting natural killer (NK) cell and chimeric antigen receptor (CAR)-redirected T-cell therapy or a combination of both (3, 6–9).

Many studies have shown in NB and other tumors that the presence of elements with immunosuppressive capacity in the tumor microenvironment (TME) must be considered a major problem responsible for the limited success of GD2-based targeted immunotherapy (10–14). In addition, previous studies have shown that NB includes two types of tumor cells: undifferentiated mesenchymal cells (MES) and committed adrenergic cells (ADRN) that can interconvert, causing extreme cellular heterogeneity. Importantly, MES cells are enriched in post-therapy and relapsed tumors and result in more chemoresistance *in vitro* (15). These cells are often characterized by a decreased GD2 expression compared with ADRN (16) indicating a possible mechanism occurring in the failure of GD2 targeting approaches. Recently, Mabe et al. demonstrated the possible mechanism of resistance to anti-GD2 therapy in NB in which the MES and the epigenetic rewiring to the ADRN state can control the levels of GD2 through the expression of *ST8SIA1* (GD3S), an upstream GD2 catalytic enzyme, by the enhancer of zeste homolog 2 (EZH2)-dependent modulation. Notably, inhibition of EZH2 restored the expression of GD3S and GD2 as well as the sensitivity to anti-GD2 monoclonal antibody both *in vitro* and *in vivo* (16).

In this context, in an attempt to integrate these important findings, we analyzed primary NB cell cultures that faithfully mimic the properties of the original tumors. For this purpose, we studied two primary NB cell cultures, WU-1 and WU-2, respectively, and two MES/ADRN primary cultures (691 and 717) derived from the same patient.

Our studies highlight four major points: i) NB-MES primary cultures still express a high level of GD2 and, upon co-culture with NK cells, undergo an editing process with the acquisition of the CD105^+^/CD90^+^/CD73^+^/TAZ^+^/GD2^bright^ mature mesenchymal phenotype (12) and a strong upregulation of GD2 expression. ii) ADRN to MES transition (AMT) is not only associated with tumor progression (1–4) but also correlated with the development of immune escape mechanisms from NK cell-mediated control. iii) In both ADRN and MES primary cultures expressing high levels of GD2, resistance to NK cell-mediated killing is overcome by the administration of dinutuximab beta, the anti-GD2 monoclonal antibody, differing from what is reported in animal models (16) and, more importantly, confirming the results obtained in phase I/II clinical trials (8). iv) ADRN primary cells inhibit NK cell functions.

## Material and methods

See **Supplementary File 1**.

## Results

### GD2 expression in mesenchymal and adrenergic primary cultures

**Figure 1:**
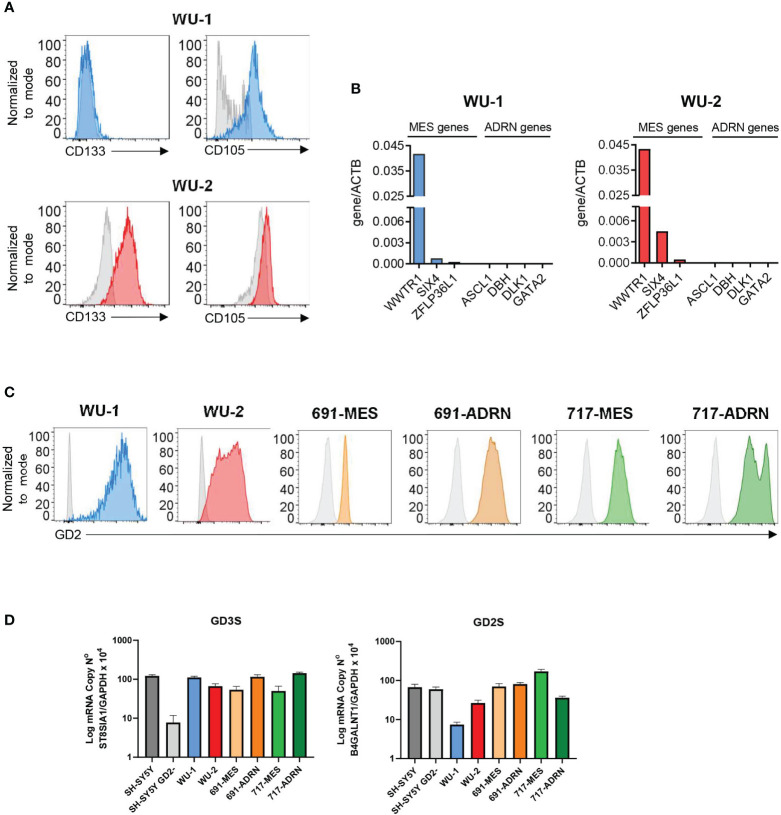
Ganglioside sialic acid-containing glycosphingolipid 2 (GD2) expression in neuroblastoma (NB) primary mesenchymal and adrenergic cultures. **(A)** Flow cytometry analysis of the surface expression of the mesenchymal markers CD133 and CD105 in NB primary cultures WU-1 and WU-2. Representative histograms of three independent experiments. **(B)** RT-PCR analysis of the NB mesenchymal transcripts WWTR1, SIX4, and ZFLP36LI and NB adrenergic markers ASCL1, DBH, DLK1, and GATA2 in NB primary cultures WU-1 and WU-2. mRNA levels were normalized with ACTB and were expressed as fold increase relative to the untreated controls. **(C)** Flow cytometry analysis of the surface expression of GD2 in mesenchymal NB primary cultures (WU-1, WU-2, 691-MES, and 717-MES) and in NB adrenergic primary cultures (691-ADRN and 717-ADRN). Representative histograms of three independent experiments. **(D)** RT-PCR analysis of the sialyl-transferase GD3 synthase (GD3S, ST8SIA1) transcript (left panel) and GD2 synthase (GD2S, B4GALNT1) transcript (right panel) in NB primary mesenchymal and adrenergic cell cultures. The adrenergic cell line SH-SY5Y and its GD2 silenced counterpart SH-SY5Y/GD2^−^ were employed as controls of expression. Results were expressed as means ± SEM (*n* = 3).

We characterized two primary NB cell lines (WU-1 and WU-2) and two pairs of MES/ADRN primary cultures derived from the same patient (691 and 717). Flow cytometry analysis (**Figure 1A**) shows that WU-1 cells exhibit a more mature CD133^−^/CD105^+^ MES phenotype that is also detected in two additional primary cultures—WU-3 and WU-4 (**Supplementary Figure 1A**), while WU-2 cells display an undifferentiated CD133^+^/CD105^−^ MES phenotype (12, 15, 16). As confirmed by the expression profile analysis, these primary cell cultures did not express genes associated with the ADRN status. On the other hand, they expressed high levels of MES markers such as TAZ (WWTR1) and SIX4 (**Figure 1B**; **Supplementary Figure 1B**) (17, 18). As shown in **Figure 1C**, both primary NB cells with either undifferentiated (WU-2) or mature (WU-1) MES phenotype expressed high levels of GD2. The analysis of two pairs of MES and ADRN primary cultures shows that GD2 was strongly expressed by ADRN ones (i.e., 691-ADRN and 717-ADRN) (16) and was still consistently preserved in their MES counterparts (i.e., 691-MES and 717-MES) (**Figure 1C**).

Subsequently, we investigated the expression of GD3 (GD3S, ST8SIA1) and GD2 (GD2S, B4GALNT1) synthases that are involved in GD2 expression (16). As shown in **Figure 1D**, all our NB primary cultures, independent on their MES or ADRN phenotype, expressed GD3S and GD2S. As a control in the RT-PCR analysis, we used the SH-SY5Y NB cell line that contained both GD2^neg^ and GD2^pos^ clones. As previously reported in the GD2^neg^ clone, the expression of GD3S was strongly decreased, while the expression of GD2S was not modified as compared with the SH-SY5Y GD2^pos^ clone (**Figure 1D**).

### GD2 expression on NB cells ameliorates the antitumor activity of NK cells

**Figure 2:**
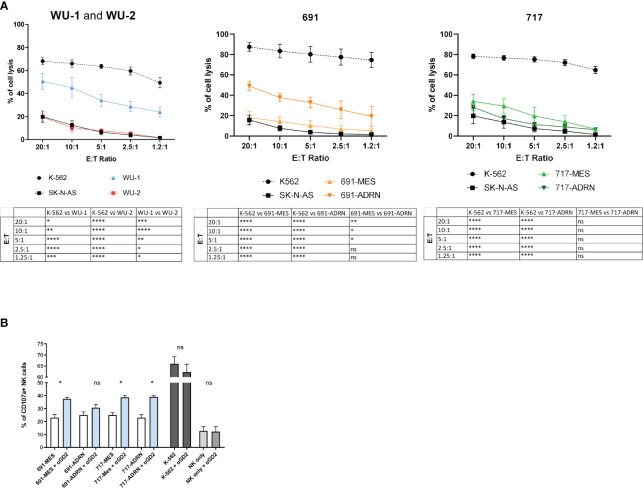
Natural killer (NK) cells mediate cytotoxic activity against NB cells. **(A)** Susceptibility of CMFDA-labeled NB primary cultures to allogenic IL-2-activated NK cell-mediated lysis at different effector:target ratios (E:T ratio). CMFDA-labeled K-562 and SK-N-AS cells were used as positive and negative lysis controls, respectively. Data were expressed as mean ± SEM (*n* = 4) of the percentage of cell lysis (propidium iodide-positive cells, PI^+^ cells). Statistical analysis was performed using two-way ANOVA with Tukey’s multiple comparisons test. *p ≤ 0.05; **p < 0.01; ***p < 0.001; ****p < 0.0001; ns, not statistically significant. **(B)** Surface expression of the degranulation marker CD107a on NK cells after co-culture with the NB mesenchymal/adrenergic pairs 691 and 717 in the presence or absence of the anti-GD2 mAb (αGD2) at 1:1 E:T ratio by flow cytometric analysis. NK cells cultured without target cells (NK only and NK only + αGD2) or with K-562 (K-562 and K-562 + αGD2) were used as internal controls. Data were expressed as the percentage of CD107a-positive cells (mean ± SEM; *n* = 4). Statistical analysis was performed using the Mann–Whitney test. *p ≤ 0.05; ns, not statistically significant.

We next analyzed the susceptibility of primary NB cells to NK cell killing. **Figure 2A** shows that WU-2 cells are highly resistant to NK cell-mediated lysis, while WU-1 cells exhibit an intermediate but still significant resistance to NK cell-mediated lysis (**Figure 2A**, left panel). This behavior is also observed in two additional MES mature primary cultures: WU-3 and WU-4 (**Supplementary Figure 1C**). Notably, 691-MES and both 717-MES and 717-ADRN cells were highly resistant, while 691-ADRN cells exhibited a partial but still significant resistance to NK cell-mediated killing (**Figure 2A**, central panels). The SK-N-AS NB cell line and the K-562 erythroleukemia cell line were used as controls. As expected, K-562 cells were highly susceptible to NK cell killing, while the SK-N-AS cell line resulted in more resistant cells.

Subsequently, we investigated whether the resistance to NK cell-mediated killing could be overcome in the presence of the anti-GD2 monoclonal antibody dinutuximab beta via antibody-dependent cellular cytotoxicity (ADCC). In the presence of an anti-GD2 monoclonal antibody, we observed a statistically significant increase in the NK cell degranulation capability (CD107a expression) in contact with both ADRN and MES (691 and 717) cells (**Figure 2B**). The fold increase observed in the presence of anti-GD2 mAbs ranged from 25.77% to 72.98% compared with the same GD2^+^ NB cells in the absence of anti-GD2 mAb (**Supplementary Figure 1D**). As control, we used K-562 cells that are highly susceptible to NK cell lysis and induce high percentages of CD107a expression when compared with primary NB cells (**Figure 2B**). Importantly, since K-562 did not express the GD2 antigen in the presence of anti-GD2 mAbs, the degranulation capability of NK cells was not modulated (**Supplementary Figure 1D**). These results indicate that the use of dinutuximab beta, independent on the ADRN or MES phenotype, induces an increase of NK-mediated antitumor activity only in GD2^+^ tumor cells.

### NK cytolytic activity was impaired by primary NB cells

**Figure 3:**
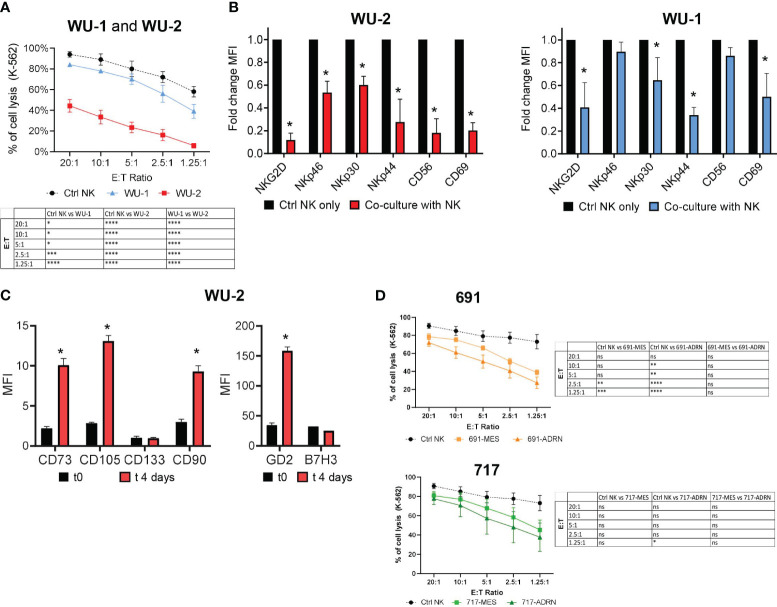
Interactions between primary mesenchymal and adrenergic cell cultures with NK cells. **(A)** Evaluation of the cytotoxic activity of freshly isolated PB NK cells against CMFDA-labeled K-562 target cells at different E:T ratios. NK cytotoxicity assay was performed after 4 days of co-culture with NB primary mesenchymal cells WU-1 and WU-2. NK cells cultured for 4 days in an IL-2-supplied medium were used as control (Ctrl NK). Data were expressed as mean ± SEM (*n* = 4) of the percentage of K-562 cell lysis (PI^+^ cells). Statistical analysis was performed using two-way ANOVA with Tukey’s multiple comparisons test. *p ≤ 0.05; **p < 0.01; ***p < 0.001; ****p < 0.0001; ns, not statistically significant. **(B)** Flow cytometric analysis of the NK cell phenotype for NKGD2, NCRs (NKp46, NKp30, NKp44), CD56, and CD69 on NK cells after co-culture with WU-1 and WU-2 NB primary cells. Freshly isolated PB NK cells were co-cultured for 4 days either in the presence of WU-1 and WU-2 primary cells cell lines or alone (Ctrl NK only) in an IL-2-supplied medium. Fold change mean fluorescence intensity (MFI) was expressed as mean ± SD (*n* = 3). *p < 0.05 sample co-culture with NK vs. Ctrl NK only. **(C)** Phenotype of WU-2 primary NB cells, after 4 days of co-culture, with freshly isolated PB NK cells by flow cytometry analysis. The MFI ratio of the indicated markers was expressed as mean ± SD (*n* = 3). *p < 0.05 sample co-culture with t0 vs. t4 days. **(D)** Percentage of CMFDA-labeled K-562 cell lysis in cytotoxicity assays using freshly isolated PB NK cells after 4 days of co-culture with NB cells 691 and 717. NK cells cultured alone were used as controls (Ctrl NK). Values are expressed as mean ± SEM (*n* = 3). Statistical analysis was performed using two-way ANOVA with Tukey’s multiple comparisons test. *p ≤ 0.05; **p < 0.01; ***p < 0.001; ****p < 0.0001; ns, not statistically significant.

Previous studies demonstrated the inhibitory potential of selected NB cell lines on the effector function of NK cells (12). Thus, we investigated whether the undifferentiated or mature status of NB cells could differently affect the NK cell antitumor responses. To this end, we co-cultured NB primary cells (WU-1 and WU-2) with freshly isolated NK cells at a 3:1 effector:target (E:T) ratio for 6 days. After co-culture, NK cells were isolated and their cytolytic activity was tested against K-562 target cells in different E:T ratios (**Figure 3A**).

Since WU-2 cells strongly inhibited the cytolytic activity of NK cells against K-562 target cells, we investigated more deeply whether this inhibitory effect was related to the expression of the major activating NK receptors. In particular, we analyzed by flow cytometry the expression of NCRs, NKG2D, and the activation marker CD69. In line with the results of **Figure 3A**, NK cells, upon co-culture with WU-2, strongly downregulated the expression of all receptors analyzed (**Figure 3B**). Conversely, the cytolytic activity of NK cells was only partially but significantly affected upon co-culture with WU-1 (**Figure 3A**), as a possible consequence of a limited downregulation on the expression of NKG2D, NKp30, NKp44, and CD69 on NK cells (**Figure 3B**).

Notably, we found upon co-culture with NK cells that the undifferentiated WU-2 cells underwent an immune editing process, acquiring a mature, mesenchymal-like phenotype (CD73^bright^/CD105^bright^/CD90^bright^), and strongly increased the GD2 expression, while B7-H3 expression, another NB marker, was not affected (**Figure 3C**). Using the same experimental setting, we also analyzed the effects of both pairs of MES or ADRN cells on NK cell antitumor activity. As shown in **Figure 3D**, both pairs of MES or ADRN cells could induce a partial decrease of NK cytolytic activity against K-562 target cells.

All these data suggested that NB is composed of a heterogeneous cell population that could modulate their phenotype from MES to ADRN and vice versa. However, GD2 expression is not affected by this phenotypic conversion and remains expressed at high levels.

## Discussion

Studies on NB immortalized cell lines (15) indicate that NB includes two types of interconverting tumor cells: undifferentiated and mature MES and committed ADNR cells. The latter ones express high levels of GD2 (13) that, owing to its restricted expression in many tumors of neuroectodermic origin, constitutes a privileged target for NB immunotherapy (2). By contrast, MES cells significantly expand in post-therapy and relapsed tumors and display a decreased expression of the GD2 antigen, thus potentially compromising anti-GD2 targeted immunotherapies (13). In order to verify these data, which have been mainly obtained with commercial cell lines, we analyzed primary NB cell cultures. Indeed, primary cancer cell cultures represent a key element in cancer research preserving the biological and molecular characteristics of the original tumor more faithfully than the commercial cell lines (19). For this purpose, we studied six NB primary cultures for the presence of mesenchymal neuroblasts, analyzing their GD2 expression and their interactions with NK cells. Remarkably, different from what was recently reported (16), we found that both undifferentiated and mature MES neuroblasts consistently express high levels of GD2 as well as of the enzymes that control GD2 expression (GD3S and GD2S), confirming that GD2 expression is a highly complex and finely regulated process (2–4).

In this context, Mabe et al. demonstrated in two different experimental murine models (16) that anti-GD2 treatment induces a selection of GD2^neg^ NB cells that become refractory to anti-GD2 targeted therapies. However, none of the high-risk relapsed/refractory NB patients who previously received dinutuximab beta and then recruited in a phase I/IIa clinical trial (NCT03373097) based on the treatment with a third-generation GD2.CAR T cells developed GD2^neg^ blasts (8). Furthermore, it emerges from the same study that no difference in terms of response (EFS and OS) can be observed between patients who received or did not receive dinutuximab beta before autologous GD2.CAR T-cell treatment. Concerning this point, we speculate that compared with the study reported by Mabe et al., mice and humans may differently control GD2 expression. Additionally, two different treatment protocols for patients with NB are in use in Europe and the USA, namely, “chemoimmunotherapy” (anti-GD2 monoclonal antibody + chemotherapy) and anti-GD2 monoclonal antibody alone (20).

Subsequently, we analyzed the interactions between the eight NB primary cultures and NK cells, and our data demonstrate that four out of six primary cultures (WU-2, 691-MES, and both 717-MES and 717-ADRN) are almost totally resistant to NK cell-mediated killing, while WU-1, WU-3, and WU-4 and 691-ADRN displayed an intermediate but still significant susceptibility to NK cell-mediated killing. The mechanisms that control NB resistance to the killing by immune cells are multiple and not yet fully elucidated (10, 12, 15, 21–24).

Notably, both GD2^pos^ ADRN and MES primary cultures were sensitive to ADCC induced by the treatment with the anti-GD2 mAb dinutuximab beta, as shown by the increased expression of the degranulation marker CD107a observed in NK cells upon contact with the abovementioned NB primary cultures. These results clearly indicate that although NB cells were resistant to NK cell killing, the expression of GD2 on these cells and the use of GD2-monoclonal antibody-based immunotherapy make them susceptible to the antitumor activity exerted by NK cells.

Finally, we analyzed the ability of the six NB primary cultures, upon co-culture with freshly isolated NK cells, to interfere with the cytolytic activity of NK cells against the K-562 target cells. WU-2 MES cells strongly downregulated by cell–cell contact mechanism the expression of several NCRs, NKG2D, and the activation marker CD69, almost completely inhibiting the cytolytic activity of NK cells against the K-562 target cells. While WU-1 mature mesenchymal cells exerted only a mild downregulation on the expression of NKG2D, NKp30, NKp44, and CD69 and partially inhibited the NK cell cytotoxic activity. Of note, we observed that both pairs of ADNR/MES subsets were able to partially inhibit the cytolytic activity of the NK cells under the condition of cell-to-cell contact. Finally, we observed that WU-2 cells, upon 6 days of co-culture with freshly isolated NK, underwent an immune editing process, acquiring the mature mesenchymal phenotype (CD105^bright^/CD90^bright^/CD73^bright^), and strongly increased their GD2 expression. Thus, it is tempting to speculate that MES may convert not only into ADNR cells but also into mature mesenchymal neuroblasts.

In conclusion, the interconversion between MES and ADNR cells can result in a high intratumoral heterogeneity generating additional subsets of neuroblasts that may express unattended properties (12, 15, 16) such as increases of immunosuppressive activity and expression of high levels of GD2 independent on their MES or ADRN phenotype, therefore behaving differently from what was reported for commercial cell lines (12, 16). More importantly, GD2-positive primary MES and ADRN cultures are resistant to NK-mediated killing. Of note, this resistance could be overcome by ADCC-mediated by anti-GD2 mAb.

## Data availability statement

The original contributions presented in the study are included in the article/**Supplementary Material**. Further inquiries can be directed to the corresponding author.

## Ethics statement

The studies involving humans were approved by the Institutional Ethical Committee of University Hospital Würzburg - Ethical Committee Approvals N°AZ250/20. The studies were conducted in accordance with the local legislation and institutional requirements. Written informed consent for participation in this study was provided by the participants’ legal guardians/next of kin.

## Author contributions

SM: Data curation, Investigation, Methodology, Writing – original draft. MTB: Data curation, Investigation, Methodology, Writing – review & editing. AP: Data curation, Investigation, Methodology, Writing – original draft, Writing – review & editing. DH: Investigation, Methodology, Writing – review & editing. TT: Investigation, Methodology, Writing – review & editing, Data curation. GW: Formal analysis, Investigation, Writing – review & editing. P-GS: Writing – review & editing. MB: Investigation, Methodology, Writing – review & editing. LM: Funding acquisition, Supervision, Writing – original draft, Writing – review & editing. EM: Writing – review & editing, Supervision. BA: Formal analysis, Supervision, Writing – review & editing, Conceptualization. PV: Funding acquisition, Supervision, Writing – original draft, Writing – review & editing. NT: Conceptualization, Investigation, Supervision, Writing – original draft, Funding acquisition, Writing – review & editing. IC: Funding acquisition, Investigation, Project administration, Supervision, Writing – original draft, Writing – review & editing, Conceptualization.

## Supplementary material

Supplementary Figure 1 Expression of mesenchymal markers in NB primary mesenchymal cultures and sensitivity to NK-mediated killing. **(A)**. Flow cytometry analysis of the surface expression of the mesenchymal markers CD133 and CD105 in NB primary cultures WU-3 and WU-4. **(B)**. RT-PCR analysis of the NB mesenchymal transcripts WWTR1, SIX4, and ZFLP36LI and NB adrenergic markers ASCL1, DBH, DLK1, and GATA2 in NB primary cultures WU-3 and WU-4. mRNA levels were normalized with ACTB and were expressed as fold increases relative to the untreated controls. **(C)**. Susceptibility of CMFDA-labeled NB primary mesenchymal cultures, WU-1, 2, 3, and 4 to allogenic IL-2 activated NK cell-mediated lysis at different effector:target ratios (E:T ratio). CMFDA-labelled K-562 were used as positive control. **(D)**. Fold increase of CD107a expression between cells in the presence of anti-GD2 mAb vs the same cells in the absence of anti-GD2 mAb.
