# Supplementary material for: Transition to a mesenchymal state in neuroblastoma may be characterized by a high expression of GD2 and by the acquisition of immune escape from NK cells
Source: Front Immunol. 2024 Apr 26;15:1382931. doi: 10.3389/fimmu.2024.1382931 (PMC11082345; doi:10.3389/fimmu.2024.1382931)
Supplement: Supplementary file 1 [file DataSheet_1.docx]

**Supplemental Material**

**Materials and methods**

**Cell lines and reagents**

SH-SY5Y, SK-N-AS, and K-562 commercial cell lines were purchased from ATCC and cultured as indicated in^(1)^.

Freshly isolated NK cells were purified from peripheral blood mononuclear cells (PBMC) obtained from anonymous healthy donors, cultured, and polyclonally activated NK as described before^(2)^.

Primary patient-derived-cell lines (WU-1, 2, 3, and 4) were derived from pediatric neuroblastoma patient tissue samples immediately after collection (biopsy or resection) and after written informed consent was obtained by the patient’s legal guardians using a previously established protocol. Patient tissues were collected in accordance with the Institutional Ethical Committee of University Hospital Würzburg - Ethical Committee Approvals N°AZ250/20.

Neuroblastoma (NB) primary cell lines (691-MES, 691-ADNR, 717-MES, and 717-ADRN) were obtained from the Department of Oncogenomics, Academic Medical Center (Amsterdam, Netherlands), and cultured as described in^(3)^.

Commercial cell lines identities were validated by Short Tandem Repeat profiling and tested negative for mycoplasma by Mycoplasma Detection Kit (Venor-GeM Advance, Berlin, Germany) before experimental use.

**Flow cytometry analysis:**

NB or NK primary cell cultures were stained to detect surface markers using fluorochrome-conjugated monoclonal antibodies (mAbs) for 20 min at 4°C, and stained as previously described(1, 2).

The following mAbs were employed:

APC Anti-human CD133 (#130-113-184), PE Anti-human CD105 (#130-112-163), VioBlue Anti-human CD90 (#130-114-866), PE Anti-human CD69 (#130-092-160) provided by Miltenyi-Biotec (Bergisch Glabdach, Germany); FITC Anti-human CD73 (#561254), BV510 Anti-human NKp30 (#743170) all provided by DB-Biosciences (San Jose, U.S.A.); PE/Dazzle594 Anti-human NKGD2 (#320828), PE/Dazzle 594 Anti-human GD2 (#320828), PE/Cy7 Anti-human B7-H3 (#351008), APC Anti-human NKp44 (#325110) all provided by Biolegend (San Diego, U.S.A); ef450 Anti-human NKp46 (#48-3359-42) provided by Thermo-Fisher Scientific (Waltham, U.S.A.) and Pe-Cy7 Anti-human CD56 (#A21692) provided by Beckman-Coulter (Brea, U.S.A.).

Primary antibodies were diluted 1:25 in 5% FBS (FBS; Euroclone) in Dulbecco’s Phosphate Buffered Saline (DPBS, Euroclone).

**Real-time qRT-PCR**

B4GALNT1 and ST8SIA1 mRNA were measured by real-time quantitative reverse transcription–polymerase chain reaction (qRT-PCR) analysis. Total RNA was isolated using the RNeasy Mini kit and QIAshredder (Qiagen, Hilden, German) according to the manufacturer’s instructions. For cDNA generation, 300 ng RNA was reverse transcribed using Vilo SuperScript cDNA Synthesis kit (Thermo Fisher Scientific, Waltham, Massachusetts, USA). Five ng cDNA were mixed with dye-labeled TaqMan primers for GAPDH, B4GALNT1, and ST8SIA1 (Hs03929097_g1, Hs00155195_m1, and Hs01124289_m1 respectively, Thermo Fisher Scientific) and TaqMan Multiplex Mastermix (Thermo Fisher Scientific) following manufacturer’s instructions. Samples were amplified on a Quantstudio 6 (Thermo Fisher Scientific) in quadruplets and interpolating the Ct values into a standard curve that generated the formula: mRNA copy number = power (10, (Ct −39.7)/− 3.47). The normalized copy number for B4GALNT1 or ST8SIA1 respectively, was determined as follows: (mRNA Copy number B4GALNT1 or ST8SIA1/mRNA copy number GAPDH)*10^4^.

The protocols utilized have been previously explained in(1).

The indicated primers were used in the study: WWTR1 (alias TAZ) fw: 5’- CCTTTTCGCCCAGCACTAGC -3’, rv: 5’- ACTCTGTGCCTGGCAGTCTA -3’; SIX4 fw: 5’- AAGGGCCCACTCTAGTTCTC -3’, rv: 5’- GGGAGAGGAATTCTTCATAGGG -3’; ZFP36L1 fw: 5’- CACTAAATTGTGCCGCGCTC -3’, rv: 5’- TGGGACGCACAGAATGTTCA -3’; ASCL1 fw: 5’- AACTTCAGCGGCTTTGGCTA -3’, rv: 5’- TTGACCAACTTGACGCGGTT -3’; DBH fw: 5’- CCCCACCGTTGTCAGCATTG -3’, rv: 5’- TGGGGATCGTCGCAGAGTAG -3’; DLK1 fw: 5’- ACTGACATTGGGGGCGACTT -3’, rv: 5’- AGACACTCGTAGCTCACCTG-3’; GATA2 fw: 5’- TACCTGTGCAATGCCTGTGG -3’, rv: 5’- TGGTGGTTGTCGTCAGTCTT -3’; ACTB fw: 5’- ACCGCGAGAAGATGACCCAGA -3’, rv: 5’- GGATAGCACAGCCTGGATAGCAA -3’.

**Cytotoxicity Assay**

The cytolytic activity of NK cells was evaluated using a flow cytometric assay to assess NK cell killing(1, 2). Primary culture cells derived from NB were utilized as targets with various Effector:Target ratios (E:T ratio). K-562 and SK-N-AS cell lines were used as positive and negative controls for cell lysis, respectively.

**Co-Culture of NK and Target Cells**

For co-culture experiments, freshly isolated NK cells were cultured at a density of 3×10^5^ cells per well in 24-well plates with 600 U/mL of IL-2 (Proleukin; Novartis-Farma, Origgio, Italy), in the presence or in the absence of NB cell lines, at 3:1 ratio (NK:tumor cells). After 6 days, the NK cells were collected and used as effectors in the cytotoxicity assays.

K-562 cells were labeled with 5 µM of Cell Tracker Green (CMFDA; Thermo-Fisher Scientific). The target cells were then co-incubated with NK cells at 37°C at different E:T ratios. After 4 hours of incubation, propidium iodide (PI, Sigma-Aldrich) was added and the cells were analyzed using the Beckman-Coulter Cytoflex-LX flow cytometer. In this cytotoxicity assay killed target cells were identified as CMFDA^+^ and PI^+^. The percentage of cell lysis was calculated as indicated in (1).

**NK cell degranulation assay**

NK cell degranulation was studied by measuring the surface expression of CD107a on NK cells. NK cells were incubated for 4 hours with NB cells at 1:1 E:T ratio in the presence or absence of anti-GD2 mAbs (dinutuximab beta Eusa Pharma, Netherlands, 100 ng/ml(4)) and in presence of ef660 Anti-human CD107a, Invitrogen, 1:25). After 1 hour of incubation, Golgi Stop reagent (BD Biosciences) was added and incubated for additional 3 hours.  Cells were analyzed using the Beckman-Coulter Cytoflex-LX flow cytometer (5).

**Statistical Analysis**

The data analysis was performed using GraphPad Prism 8 Software (GraphPad Software 8.0.1) for statistical purposes. To assess statistical significance, a two-tailed paired Student's t-test was applied with Bonferroni correction for multiple comparisons and 2-way ANOVA with Tukey's multiple comparisons test. The results were expressed as the mean ± standard error medium (SEM), and statistical significance was defined * p≤ 0.05; **p<0.01; ***p<0.001; ****p<0.0001; ns=not statistically significant.

**References**

1. Canzonetta C, Pelosi A, Di Matteo S, Veneziani I, Tumino N, Vacca P, et al. Identification of Neuroblastoma Cell Lines with Uncommon Taz(+)/Mesenchymal Stromal Cell Phenotype with Strong Suppressive Activity on Natural Killer Cells. *J Immunother Cancer* (2021) 9(1). doi: 10.1136/jitc-2020-001313.

2. Di Matteo S, Avanzini MA, Pelizzo G, Calcaterra V, Croce S, Spaggiari GM, et al. Neuroblastoma Tumor-Associated Mesenchymal Stromal Cells Regulate the Cytolytic Functions of Nk Cells. *Cancers (Basel)* (2022) 15(1). doi: 10.3390/cancers15010019.

3. van Groningen T, Koster J, Valentijn LJ, Zwijnenburg DA, Akogul N, Hasselt NE, et al. Neuroblastoma Is Composed of Two Super-Enhancer-Associated Differentiation States. *Nat Genet* (2017) 49(8):1261-6. doi: 10.1038/ng.3899.

4. Barry WE, Jackson JR, Asuelime GE, Wu HW, Sun J, Wan Z, et al. Activated Natural Killer Cells in Combination with Anti-Gd2 Antibody Dinutuximab Improve Survival of Mice after Surgical Resection of Primary Neuroblastoma. *Clinical cancer research : an official journal of the American Association for Cancer Research* (2019) 25(1):325-33. doi: 10.1158/1078-0432.CCR-18-1317.

5. Cantoni C, Serra M, Parisi E, Azzarone B, Sementa AR, Nasto LA, et al. Stromal-Like Wilms Tumor Cells Induce Human Natural Killer Cell Degranulation and Display Immunomodulatory Properties Towards Nk Cells. *Oncoimmunology* (2021) 10(1):1879530. doi: 10.1080/2162402X.2021.1879530.
