# Supplementary figures and images for: Transition to a mesenchymal state in neuroblastoma may be characterized by a high expression of GD2 and by the acquisition of immune escape from NK cells
Source: Front Immunol. 2024 Apr 26;15:1382931. doi: 10.3389/fimmu.2024.1382931 (PMC11082345; doi:10.3389/fimmu.2024.1382931)

A

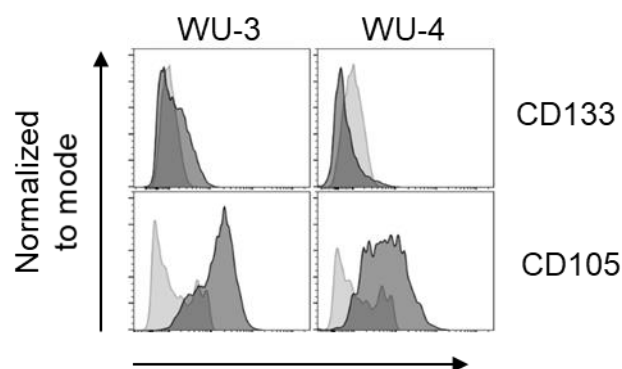

B

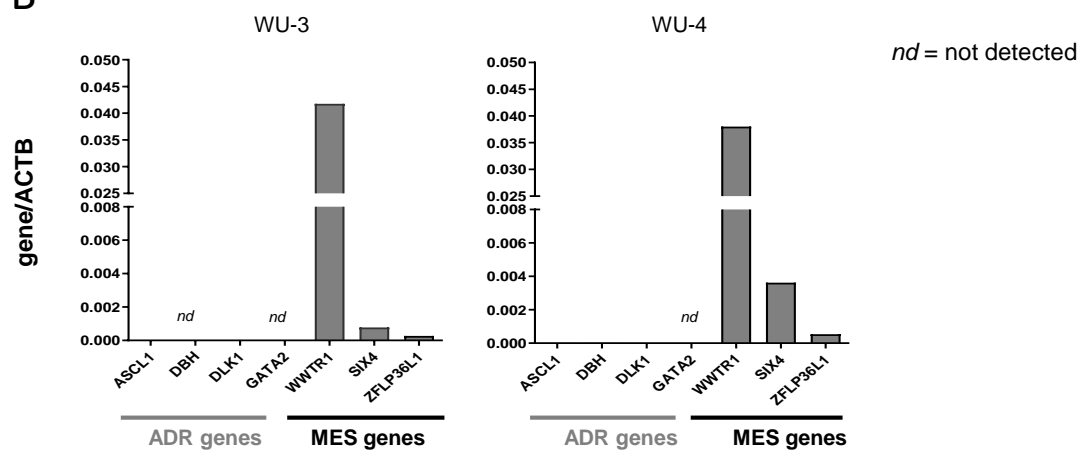

C

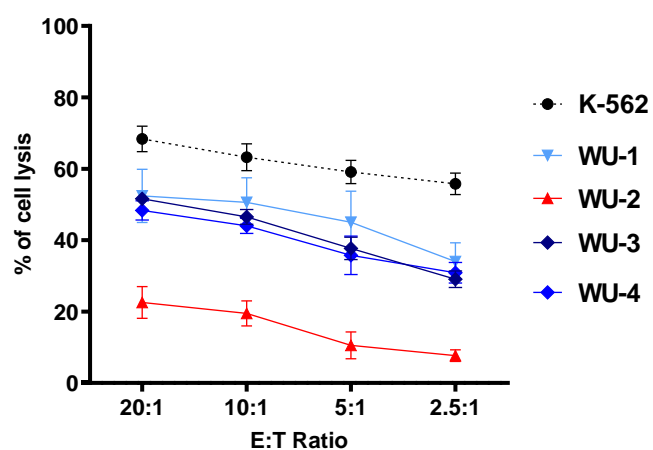

D

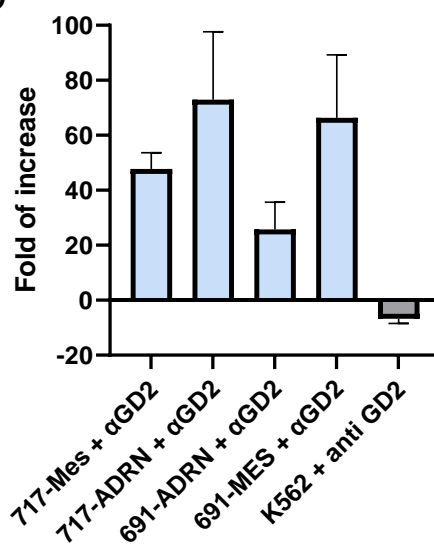

Supplementary Figure 1

Supplement: Supplementary Figure 1 — Expression of mesenchymal markers in NB primary mesenchymal cultures and sensitivity to NK-mediated killing. (A). Flow cytometry analysis of the surface expression of the mesenchymal markers CD133 and CD105 in NB primary cultures WU-3 and WU-4. (B). RT-PCR analysis of the NB mesenchymal transcripts WWTR1, SIX4, and ZFLP36LI and NB adrenergic markers ASCL1, DBH, DLK1, and GATA2 in NB primary cultures WU-3 and WU-4. mRNA levels were normalized with ACTB and were expressed as fold increases relative to the untreated controls. (C). Susceptibility of CMFDA-labeled NB primary mesenchymal cultures, WU-1, 2, 3, and 4 to allogenic IL-2 activated NK cell-mediated lysis at different effector:target ratios (E:T ratio). CMFDA-labelled K-562 were used as positive control. (D). Fold increase of CD107a expression between cells in the presence of anti-GD2 mAb vs the same cells in the absence of anti-GD2 mAb. [file Image_1.pdf]
